# Improving the performance of mini-channel heat sink by using wavy channel and different types of nanofluids

**DOI:** 10.1038/s41598-022-13519-0

**Published:** 2022-06-07

**Authors:** Zahraa H. Saadoon, Farooq H. Ali, Hameed K. Hamzah, Azher M. Abed, M. Hatami

**Affiliations:** 1 College of Engineering, Mechanical Engineering Department, University of Babylon, Babylon City, Iraq; 2 Air Conditioning and Refrigeration Techniques Engineering Department, Al-Mustaqbal University College, Babylon, 51001 Iraq; 3 Department of Mechanical Engineering, Ferdowsi University of Mashhad, Mashhad, Iran

**Keywords:** Nanoparticles, Applied mathematics, Mechanical engineering

## Abstract

The combination of nano fluid and changing cross-section mini-channel heat sink effects have become a remarkable choice for the use of thermal devices such as miniature electronic devices to be effectively cooled. In this paper, the comparison of three dimensional straight and wavy channel configuration with using different types nano fluids are numerically investigated. The effects of wave amplitude and A particular type of volume fraction of (Copper Oxide CuO, Dimond Al_2_O_3_, Iron Oxide Fe_3_O_4_, Titanium Oxide TiO_2_ and Silver Ag-nano fluids are offered. Three amplitudes of waves (0.15 mm, 0.2 mm and 0.25 mm) and Reynold’s number from 200 to 1000 and concentration volume varieties from 0 to 0.075 are used. The effect on thermal resistance, pressures drop, factor of friction of the mini channel is displayed. It is observed that the mini-channel sink's heat transfer efficiency is greatly enhanced compared to the straight channel in an event of adding distilled water as accoolant. The results indicate that nano fluid and wavy mini-channel can boost the heat sink's hydrothermal efficiency and Ag- water nano fluid in term of heat transfer, it outperforms other nanofluids an enhancement in the Nusselt number reached to 54% at concentration volume 0.075.

## Introduction

In the last quarter of the last century, the invention of microelectronic devices brought a revolution in the industry of electronics in 1965, Moore has seen this thumbnail and showed that "every two years" The Transistor Number in an integrated circuit doubled and predicted it to continue in the future. In recent decades, constraints on traditional energy sources and environmental pollution issues have prompted engineers to recover the efficiency of thermal systems, as these devices produce heat during their operation and must be continuously extracted for the sake of their effective and reliable operation.

For that purpose a heat sink is used, as air cool heatsinks are The most widely used electronic processor cooling equipment, and due to the low thermal conductivity and air heat capacity, These systems cannot cool fast processors of a minor dimension and as a consequence the heat flow is very high. Although liquid-cooled heatsinks have superior performance compared to air, improving the performance of these dispersants has attracted the attention of researchers, as traditional working fluids are characterized by low thermal performance, so it is necessary to use fluids with better thermal properties instead of conventional liquids are known as nano fluid that have higher thermal conductivity from conventional liquids, therefore, the dispersion of solid particles in base fluids may rise a thermal properties of the main fluid, as recent studies focused on enhancing heat transfer using nanoscale liquids, as experimental and analytical studies have shown that the thermal conductance of nanoscale fluids is larger than conventional liquids and thus they are more efficient in cooling devices.

The effect of using nano fluids as a coolant have been a numerically investigated by Mohammed et al.^1^ on fluid flow and heat.transfer features in a rectangular formed microchannel heat sink (MCHS). Aluminium oxide with water are used as a coolant fluid. The result showed that the heat transfer coefficient and wall shear stress are increased when the nanoparticles volume fraction is increased although the heat sink 's thermal resistance is reduced.

The rectangular, trapezoidal, and triangular micro channel heat sinks were numerically investigated by Gunnasegaran et al.^2^. The result showed the higher coefficient of heat transfer can be attained in heat sinks with a small hydraulic diameter. Water was used as a coolant fluid in three dimensional geometry.

Farsad et al.^3^ presented a numerical study of micro channel heat sink (MCHS) made from copper by using three types of nanoparticales (Al_2_O_3_–H_2_O, CuO–H_2_O and Cu–H_2_O) nano fluids as a coolants. The outcomes showed that the cooling competence of a (Al_2_O_3_/H_2_O) nano fluid micro channel heat sink (0.08) is improved by around 4.5 percent compared to distilled water micro channel heat sinks. Also, due to high thermal conductivity of pure matlic nanomaterial produce high thermal enhancement than Oxid matlic nanomaterial.

Ho and Chen^4^ studied experimentally a thermal performing of (Al_2_O_3_/H_2_O) nano fluid as a cooling fluid in a rectangular shape mini channel heat sink. The results exhibited that the nano fluid cooled heat sink has meaningfully higher average heat transfer coefficients and later overtakes the water.cooled heat sinks.

The thermal efficiency of a rectangular minichannel heatsink with (Al_2_O_3_–H_2_O) nanofluid as a working fluid in place of pure water was experimentally examined by Sohel et al.^5^. The result displayed that the heat transfer coefficient was improved by up to 18 percent. The nano fluid importantly decreased the base temperature of the heat sink (around 2.7 °C) to the pure water.

Xia et al.^6^ studied numericallythe flow of fluids and the transfer of heat in microchannel heat sink with dissimilar inlet/outlet positions (I, Z and C-type). Water was choosen as a working coolant fluid. The outcomes showed that the uniformity of flow velocity is relatively better for I-type and poor for Z-type.

The wavy microchannel heatsink and nano fluid application were numerically studied by Sakanova et al.^7^. The diamond alumina with pure water was utilized as a coolant. A three dimentional geometry with an upper and lower parallel wavy wall was investigated. The result showed that the effect of the wavy wall improves heat transfer moer clearly compared with dimond alumina mixture. Sivakumar et al.^8^ studied experimentally forced convection heat transfer performance in (Alumina Al_2_O_3_ and Copper oxide CuO–H_2_O) nano fluids in a serpentinee-shaped microchannel heatsink. The results presented that the heat transferr coefficient of CuO/water nano-fluid has enlarged compared to Al_2_O_3_–H_2_O and distilld water.

Li et al.^9^ examined the improvement of the transfer of heat and the entropyegeneration of laminar convection flow of Al_2_O_3_–H_2_O nano fluids in micro channels with flowcontrol instruments (cylinder, rectangular, protrusion, and v-groovee). The outcome of this investigation showed that the relative friction factor f/f0 of the rectangle devices micro channel is significantly larger than other shapes.

Liu et al.^10^ studied numerically the behavior of minichannel heatsinks with inlets that are not uniform. They showed a behavior of minichannel heatsinks can be influenced really by redistributing fluid flow and the use of non-uniform baffles can cause a decrease in the overall thermallresistance of minichannel heatsinks ranging between 9.9 and 13.1 percent.

Zhang et al.^11^ examined experimental work the transfer of heat and pressuredrop features by using two heat transfer improvement methods (passive-micro-fin construction and active-nano fluids) in multi-port mini channel flat tube (MMFT). The study showed that the Nusselt number rises up to 158% at the value of Reynolds number equal to 3600.

A new design (double-layer arranged) heat sink was suggested by Tang et al.^12^. The results of their study show that the double-layer structure rises heat exchange in both horizontal and vertical directions, and therefore provides an equal distribution of temperature and a great efficiency of heat transfer.

The effects of CuO–H_2_O nano fluid on a cooling performing of a two cross sectional heat sinks (rectangular and circular-cross sectional shaped) was numerically studied by Ghasemii et al.^13^. The result of a contrast of circular and rectangular channels at the similar Reynolds number attendances that the heat sink with rectangular-channelhas smaller thermal resistance.

Feng et al.^14^ carry out a numerical investigation to verify a laminar liquid flow's performance and transfer of heat in a rectangular micro channel heat sink supplied with a wire coil inserted. The conclusions presented that the transfer of heat effectiveness of a micro channel heatsink is much increased because of a longitudinal vortexes created by wire coils, However, flow resistance is augmented at the same time.

Abdollahi et al.^15^ numerically observed a heat transmission and fluid flow features of laminar nano fluid flow in micro channel heatsink with (V) type inlet/outlet prearrangement using different oxide nano fluids in water base fluid (SiO_2_, Al_2_O_3_, ZnO and CuO). The outcomes displayed that, in comparison with other studied nano fluids, the SiO_2_ nano fluid has a highest transfer rate of heat.

The flow properties and heat transmission in a cylindrical heat sink were examined by Sobamowo et al.^16^ they exposed that a decrease in a (channel helix,angle and an increase in a channel’s aspect ratio can improve the average heat transfer coefficient and pressure drop in a heat sink. Khodabandeh and Abbassi^17^ demonstrate numerically the thermal efficiency of the heatsink with a trapezoidal microchannel with lateral angles (75°, 60°, 45°, 30°). Use Al_2_o_3_–H_2_O as a coolant. It turns out that the finely deflected channel with an angle of 75° has the highest amount of Heat transfer.

The effects on a convection heat transfer coefficient, base temperature, thermal resistance and concentration volume have been documented by Saeed and Kim^18^ their results have shown that, relative to distilled water, the convection heat transfer coefficient increases substantially when using alumina nano fluids.

The thermal analysis of various geometries of mini-channel heat sink rectangular, circular, trapezoidal and square minichannels was investigated experimentally by Sinks et al.^19^. The study showed that, relative to other types of mini-channels, the pumping power needed for circular mini-channel geometry is maximum and minimum for rectangular mini-channels.

Three techniques for enhancing heat transfer were examined experimentally by Naphon et al.^20^; micro channel heat sink, nano fluids and an impact of jet. The results displayed that a suspension of nano particles in a base fluid significantly increases the transfer of convective heat at 0.015% nanofluid intensity by 18.56 percent. Furthermore, The coefficient of heat transfer produced tends to increase with increased diameter of nozzle and a reduction in the height of the nozzle.

Ambreen et al.^21^ numerical estimated a thermo fluid properties of a heat sink containing 72 fins with a circular cross section, where the Al_2_O_3_-water nanofluid was used as a coolant. The above investigation established that the addition of nanoparticles to the basic liquid increased the rate of improvement in the heat transfer coefficient by (8.4, 11.5, 16) percent to the volumetric concentrations (0.25, 0.5, 1)% respectively. Kumar and Sarkar^22^ analyze experimental and numerical work of the transfer of heat and pressure reduction characteristics of a heat dispersion consisting of 9 parallel, rectangular channels, use (Al_2_O_3_–TiO_2_) nano fluid as a coolant. The numerical and experimental outcomes exhibited that the pressure and the friction factor decrease increases by increasing in the volumetric concentration of nanoparticle, and mixing particles of different type, similar shape and size does not result an obvious effect on a rate of transfer of heat.

Sajid et al.^23^ investigated an experimental study on a heat transfer and hydrodynamic feature of TiO_2_ nano fluid as a coolant in wavy channel. The outcomes showed that nano fluids showed better heat transfer features than pure water for all type of heat sinks. The greatest increase in Nusselt numbers is recorded as 40.57 percent, by using 0.012% concentration from TiO_2_ nano fluids.

A new channel design was suggested by Abdulqadur et al.^24^ to improve the efficiency of a cylinder-shaped minichannel heat sink with a slight potential pressure drop. The concept was straight-wavy hybrid channel in which the direction of the channel vagaries from the entry straight to the wavy path. The outcomes showed that under similar operating conditions, the general output of a cylindrical mini-channel heat sink with a straight, wavy channel is higher than that with a straight channel. Experimental and numerical work was examined using water as a coolant fluid.

The thermal efficiency of Alumina Al_2_O_3_/water nano fluid movements through a rectangular micro-channel heatsink with a continuous heat flux was analyzed numerically by Kahani^25^ The result exposed that the decrease of the diameter of the nanoparticles increases the Nusselt number. At Reynolds number 100 for 1 percent volume concentration of nano fluid flow, a maximum improvements in the Nusselt number reched to the 38 percent at these conditions.

The influence of slab thickness on a total efficiency of the water mini-channel heat sink have been examined numerically by Tariq et al.^26^. The result displayed that the transfer of heat reduced, Whereas a base temperature rises and a pressure decreases, with a slab thickness (0.2 to 1.6) mm in a mini-channel.

The hydropower and thermal efficiency of a curvy channel and nano-fluids as a coolant was numerically investigated by Naranjani et al.^27^. As the coolant, water-based nano-fluids containing Al_2_O_3_ nanoparticles with volume fractions less than 4 percent were used. Reasechers showed when wavy channels are used in place of standard channels in a heatsink, the heat transfer rises by (24–36)%, while a Pumping capacity rises to 31 percent, resultant in an overall performance development of 16–24% percent.

Ataei et al.^28^ investigated an experimental study of heat transfer and thermal efficiency of rectangular mini-channel heat sink. The ranging of Rynolds number between 400 and 1000. The consequence presented that by using a hybrid-nanofluid Al_2_O_3_/TiO_2_–H_2_O in place of distiled water, the maximum heat transfer coefficient improved to 16.97% and the temperature of wall lowered to 5 °C at minimum Rynolds number. An another study, using alumina and titanium oxide nanomaterial separately. The heat transfer and pressure drop in a mini-channel heat sink have been studied by Sadegh Moghanlou et al.^29^. The outcome of this study showed that a 9.30 percent heat transfer improvement was observed with only dispersion of 0.5 vol percent Al_2_O_3_ nanoparticles. The (TiO_2_–water) was also collected to demonstrate a 4.56 percent enhancement in heat transfer.

The hydraulic and thermal features of Fe_3_O_4_-water nano fluids flowing round a heated circular cylinders with perpendicular fin in a heat sink have been experimentally studied by Qi et al.^30^. In their study, researchers found that the most acceptable working conditions for the greatest heat exchange output are nanoparticle mass fraction equal to 0.4 percent and fin height H equal to 3 mm and showed that thermal efficiency increases with fin height.

A new 4-channel (4-mm wide and 3.5-mm-depth) concentrated thermal heat sink geometry was examined with alternative flow passages by Jilte et al.^31^. Its findings showed a higher rejection rate for cooled heat sink (Al_2_O_3_) compared to pure water and an increase of 2% and 17% for a fraction of 0.5% and 5% volume respectively. The values of heat flux are 50 W and 70 W for fluid flow ranges between 30 and 180 mL/min.

Coşkun and Çetkin^32^. the study deals with each the pin–fin and nanomaterial separately, a numerical study have been examined by using the properties of nanopartical from previous experimental work. Their outcome showed that the total thermal conductance is maximized by insert micro pin fins and use a definite fraction volume ratio of nanofluids. Muhammad et al.^33^ numerical investigation of mini channel (Converging–diverging longitudinally) with different nanoparticles (Alumina, Silica and copper), the concentration ranged between 0 to 0.8 percent and Reynolds between 200 and 2300 with heat flux equal to 45Kw/m^2^. Alumina nanoparticles have maximum heat transfer rate than others two nanoparticle types.

Naphon et al.^34^ an experimental was tested for mixture water–TiO_2_ nanofluid to the rectangular minichannel heat sink made from aluminium which have different height. High improvement of the heat transfer with approximetly the sam pressure drop, the result showed for the nanofluids compared to the de-ionized pure water. For the same authors Naphon et al.^35^ and for same geometry of rectangular minichannel was studied experimentally and numerically in the case of turbulent regime and two phase nanofluid model. Results showed that the volume of fraction with two phase model more accurate than single phase model. Naphon et al.^36^ using ANN technique (artificial neural network) to simulate heat and friction effect in the heat exchanger of double tube horizontal orientation. The results obtained from ANN simulation compared with experimental results, high accuracy was obtained with error range between $\pm \, 2.5\%$ to $\pm \, 7.5\%$.

Jet impingement of different conditions are studied by Naphon et al. In^37^, numerical approach for two phase model of TiO_2_ nanoparticals of 0.2 perecent intensity. In^38^, experimental work to indicate the influence of space plate to diameter jet ratio. In^20^ Flow jet impingement properties studied experimentally on microchannel heat sink, many parameters are taken into account such as volume fraction, diameter of the nozzle, space between nozzle and heat sink and the florate of mass. In^39^ ANN technique with CFD are used to simulate jet impingement in the microchannel heat sink. In^40^ Different shapes of pin–fin (circular, conical and rectangular) was investigated experimentally. Results show that circular pin has a higher thermal efficiency than conical and rectangular by 25 percent an 12 percent respectively.

Another technique of cooling is pulsating, which are studied by Naphon et al. in two papers. The first one in^41^ where authors used ANN to simulate the fluid flow and heat transfer tube with spirally coiled under magnetic field. The numerical and experimental results compared between them produce an error range between $\pm \, 0.025$ to $\pm \, 0.05$. In the second paper, Naphon et al.^42^ used a different approach called ANFIS (adaptive neuro-fuzzy inference system) to imitate fluid and heat in helical wavy tube under magnetic field. High accuracy obtained when compared numerical and experimental data.

Line et al.^43^ investigated numerical study of wavy microchannel using finite volume approach. Three variables configurations are examined, wavelength, amplitude and together wavelength and amplitude. The results outcome show improvement performance for three configurations compared to conventional rectangular microchannel. Mustafa et al.^44^ examined the experimental and numerical work. A comparison between three different cylindrical microchannel, the first on having straight, second having parallel wavy and third having helical configuration. Results show that the helical shape gives better hydrothermal performance than wavy and straight shapes.

Shahd et al.^45^ conducted a numerical study to compare between wavy and flat cross sectional area of circular cylindrical heat sink in laminar region. Copper Oxide with water was used as a working fluid. Results showed that wavy surface reduces the wall temperature of the heat sink to 20.47 °C than wall temperature of the flat heat sink.

A comparison between straight and converge-diverge mini-channel cross sectional area was studied numerically by Zahraa et al.^46^. Two types of nanofluid are Fe_3_O_4_ and Ag are used mixed with water as coolant fluid. The simulation was made utilizing finite element approach.

According to the preceding literature review, the rate of heat transfer in a minichannel heat sink is highly reliant on the channel geometry and kind of coolant employed. Investigators have widely improved rectangular, triangular and circular channel heat sinks. Wavy channel heat sinks, however, are still at an investigational stage of understanding and minimal literature for their hydraulic and thermal efficiency is available. There are three papers published before dealing with study of fluid flow and heat transfer inside wavy minichannel, they are references 23 and references 27 and 44. In reference 23, the experimental study with parallel wavy wall was investigated. In reference 27 and 44 a numerical of FVM study of compact heat sink with a set of parallel wavy where the walls parallel to flow having parallel identical paths. In the present work, the comparison of the heat transfer rate, fluid flow structure and friction factor in one specific channel between rectangular and wavy was examined in three dimensional geometry. The wavy channel in this work is not parallel, so that the top in the wall correspond to the top in the parallel wall and the bottom in the wall corresponds to the bottom in the parallel wall, therefore, the flow in the wavy channel exposed to the narrow and expansion areas periodically. The purpose of this research is to demonstrate the impact of the wave of channel walls in two different directions on improving the thermal transfer of a mini-channel heat sink by the use five types of nano fluids in addition to distilled water as a working fluid for different concentration volume and different wave amplitudes at different flow rates and compare it with conventional channel.

## Physical model and governing equations

**Figure 1:**
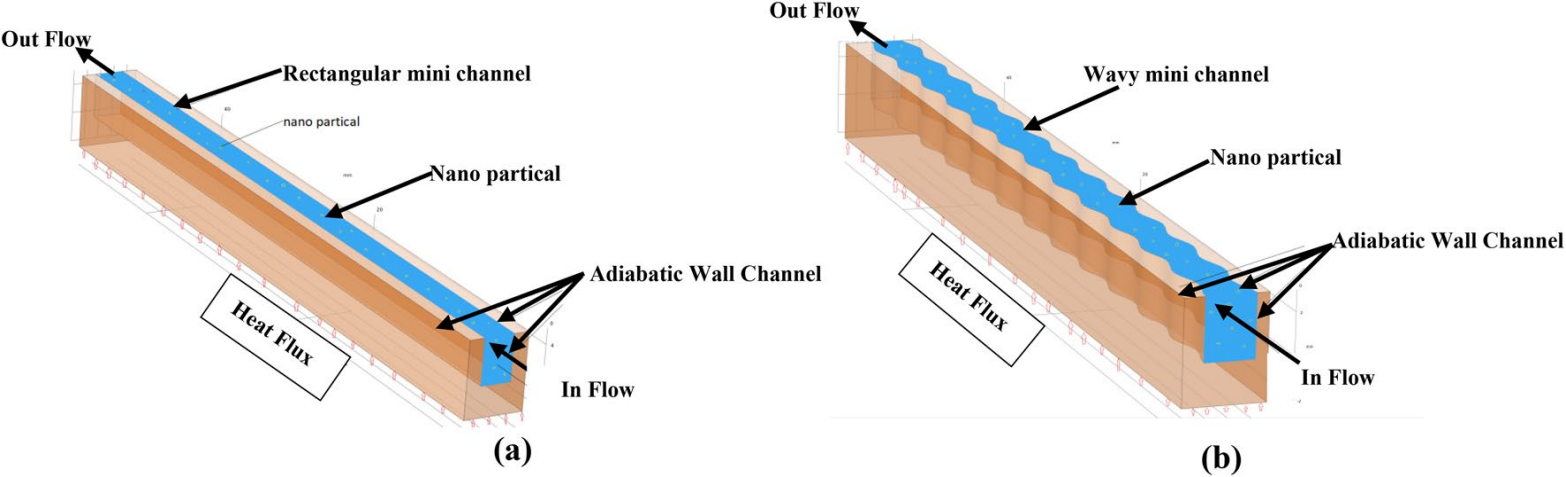
Schematic diagram of a present problem (**a**) conventional channel (**b**) wavy channel.

The physical models used in the numerical analysis are shown in Fig. 1. The heat sink with rectangular (conventional) MCHS is utilized as a standard heat sink for mini-channels. It is composed of copper and has only one channel. The geometrical parameters of conventional minichannel are the following: thickness of the walls (t) = 1 mm; width of channel (W_c_) = 2 mm; width of the heatsink (W) = 4 mm; heigh of channel (H_c_) = 3 mm; heigh of heatsink (H) = 5 mm. The lower sizes are (4 mm × 50 mm) and a uniformmheat flux 180 kW/m^2^ is obtained as shown by Fig. 1a from the lower surface.

Figure 1b shows the wavy minichannel heatsink. It has the same dimensions as a rectangular mini channel heatsink. The one change is a path of the flowing of fluid is in a cosine curve expressed by following equation:

1 $$X=A*(1-\mathrm{cos}\left(2*N*\pi *S\right))$$

where Y = S, 50 N$\ge S\ge 0$ = 0.25 (mm), A: Wavy Amplitude = 0.15, 0.2 and 0.25 (mm).

### Assumption

In this work, three dimensional geometry with two computational domain was explored to show the effect of variables on the heat transfer rate and fluid flow structure clearly. The first domain is the fluid computational domain represent the region where the fluid flow and convection heat transfer mode, while the second domain is the solid computational domain where represent conduction heat transfer mode, where made from copper. For both domain, approperiat assumptions and boundary conditions are developed with high accuracy to obtain a logical and correct solution. A number of assumptions about the operational condition of a minichannel are made in this study to simplify the analysis:

- The flow of working fluid is laminar, steady state, incompressible and single phase flow across a channel (where the fluid is only in its liquid state)
- The force of gravity is insignificant.
- Heat flux was constant, supplied to the base of heatsink.
- A mini channel heat sink's surface is effectively insulated.
- The mode of heat transmission, radiation is regarded as negligible.

#### Equations of governance

In this work, three dimensional geometry of the conjugate problem (fluid–solid problem) was presented. On the basis of the above assumption, the governing equation for this model is as follows:

(Continuity). (mass conservation) equations for the fluid (a coolant)^43,44^:

2 $$\frac{\partial {u}^{*}}{\partial x}+\frac{\partial {v}^{*}}{\partial y}+\frac{\partial {w}^{*}}{\partial z}= 0$$

Momentum equations in *x*, *y*, and *z* Cartesian coordinates, given as:

3 $${u}^{*}\frac{\partial {u}^{*}}{\partial x}+{v}^{* }\frac{\partial {u}^{*}}{\partial y}+{w}^{* }\frac{\partial {u}^{*}}{\partial z}=-\frac{1}{\rho } \frac{\partial p}{\partial x}+\frac{\mu }{\rho }\left(\frac{{\partial }^{2}u*}{\partial {x}^{2}}+\frac{{\partial }^{2}u*}{\partial {y}^{2}}+\frac{{\partial }^{2}u*}{\partial {z}^{2}}\right)$$

4 $${u}^{*}\frac{\partial {v}^{*}}{\partial x}+{v}^{* }\frac{\partial {v}^{*}}{\partial y}+{w}^{* }\frac{\partial {v}^{*}}{\partial z}=-\frac{1}{\rho } \frac{\partial p}{\partial y}+\frac{\mu }{\rho }\left(\frac{{\partial }^{2}v*}{\partial {x}^{2}}+\frac{{\partial }^{2}v*}{\partial {y}^{2}}+\frac{{\partial }^{2}v*}{\partial {z}^{2}}\right)$$

5 $${u}^{*}\frac{\partial {w}^{*}}{\partial x}+{v}^{* }\frac{\partial {w}^{*}}{\partial y}+{w}^{* }\frac{\partial {w}^{*}}{\partial z}=-\frac{1}{\rho } \frac{\partial p}{\partial z}+\frac{\mu }{\rho }\left(\frac{{\partial }^{2}w*}{\partial {x}^{2}}+\frac{{\partial }^{2}w*}{\partial {y}^{2}}+\frac{{\partial }^{2}w*}{\partial {z}^{2}}\right)$$

The equations of energy for the fluid (a coolant):

6 $${u}^{*}\frac{\partial {T}_{f}}{\partial x}+{v}^{* }\frac{\partial {T}_{f}}{\partial y}+{w}^{* }\frac{\partial {T}_{f}}{\partial z}=\frac{{k}_{f}}{\rho {c}_{p}}\left(\frac{{\partial }^{2}{T}_{f}}{\partial {x}^{2}}+\frac{{\partial }^{2}{T}_{f}}{\partial {y}^{2}}+\frac{{\partial }^{2}{T}_{f}}{\partial {z}^{2}}\right)$$

The equations of energy for the solid domain:

7 $$ks\left(\frac{{\partial }^{2}Ts}{\partial {x}^{2}}+\frac{{\partial }^{2}Ts}{\partial {y}^{2}}+\frac{{\partial }^{2}Ts}{\partial {z}^{2}}\right)=\boldsymbol{ }\boldsymbol{ }0$$

#### Boundary conditions

- Velocity at inlet was given by appropriate profile having maximum value (u_in_), while another velocity components are equal to zero.
- The temperature of a coolant fluid inlet (293 k).
- Pressure at exit is performed with 0 Pa.
- Constant heat flux 180 kW/m^2^ received at a base of the channel.
- There was no heat loss on any of the external surfaces.

By giving appropriate boundary conditions for velocity and temperature in the inlet section. Based on these values at inlet section, the governing equations, continuity, momentum an energy are solved.

#### Data reduction

Reynolds number Re, hydraulic diameter (D_h_) is defined as follows:

8 $$Re=\frac{\rho {u}_{in {D}_{h}}}{\mu }$$

9 $$Dh=\frac{2WsHs}{WsHs}$$

The pressure drop (Δp) between the minichannel heatsink's intake and output, friction factor (f), thermal resistance (R_th_) are determined by^14^:

10 $$\Delta p=pin-pout$$

11 $$f=\frac{\Delta \mathrm{pDh}}{2\rho {{u}_{in}}^{2}L}$$

12 $${R}_{th}=\frac{{T}_{max}-{T}_{in}}{Q}$$

where, $pin$ and $pout$ are static pressure at an entry and leaving of mini-channel heat sink, Q: total heat transfer, $Q={q}_{in}*{A}_{s}$; where q_in_ is the heat flux on the lower surface of heat sink; As is the mini channel heat sink area at the base and is expressed as A_s_ = W × L.

The Nusselts number Nu is calculated by^14^:

13 $$Nu=\frac{Q {D}_{h}}{ {k}_{f} {A}_{ht\left[{T}_{wm}-{T}_{f}\right]}}$$

where, *k*_*f*_: The fluid thermal conductivity, T_wm_: mean heat sink temperature, A_ht_ : a contact surface area of the working fluid and heat sink mini-channel and is expressed, Tout: The temperature of fluid at the outlet.

#### Thermophysicall properties of the working fluid

The working fluids used in the present study are [(Al_2_O_3_–H_2_O), (CuO–H_2_O), (TiO_2_–H_2_O), (Fe_3_O_4_–H_2_O) and (Ag–H_2_O)] nanofluids a thermophysical properties nanoparticles obtainable in in Table 1.

**Table 1:** Thermophysicallproperties of nanoparticles.

Property	Dimond (Al_2_O_3_)^7^	CuO ^7^	TiO_2_^25^	Fe_3_O_4_^31^	Ag^30^	Distilled^25^ water
Density (kg/m^3^)	3510	6500	4170	5200	10,500	997.1
Specific heat (J/kg K)	497.26	535.6	711	670	235	4179
Thermal conductivity (W/mK)	1000	20	11.8	6	429	0.613

The thermophysical characteristics of nanofluids were estimated using the following relationships:

The density and specific heat of nano fluids was calculated using Sakanova et al.^7^ model:

14 $${\rho }_{nf}=\left(1-\varphi \right){\rho }_{f}+\varphi {\rho }_{p}$$

15 $${cp}_{nf=}\frac{\varphi {\left(\rho cp\right)}_{p}+\left(1-\varphi \right){\rho }_{f}{cp}_{f}}{{\rho }_{nf}}$$

The thermal conductivity and viscosity of the nanofluids were calculated using the model^7^:

16 $${k}_{nf= }\frac{{k}_{p }+\left(n-1\right){k}_{f}-\left(n-1\right)\varphi \left({k}_{f}-{k}_{p }\right)}{{k}_{p }+\left(n-1\right){k}_{f}+\varphi \left({k}_{f}-{k}_{p }\right)}{k}_{f}$$

17 $${\mu }_{nf= \frac{{\mu }_{f}}{{\left(1-\varphi \right)}^{2.5}}}$$

## Numerical method and verification

In the current work, the Comsol Multiphysics the program was used to simulate and solve three-dimensional heat and flow problems in mini channel.

A CFD module in Comsol Multiphysics built upon a finite element method with a Galerkin aapproach to solve the partial differential equations governing the problem domain (continuity, momentum and energy for solid and fluid domains).

### Sensitivity of the mesh and validation of code

**Figure 2:**
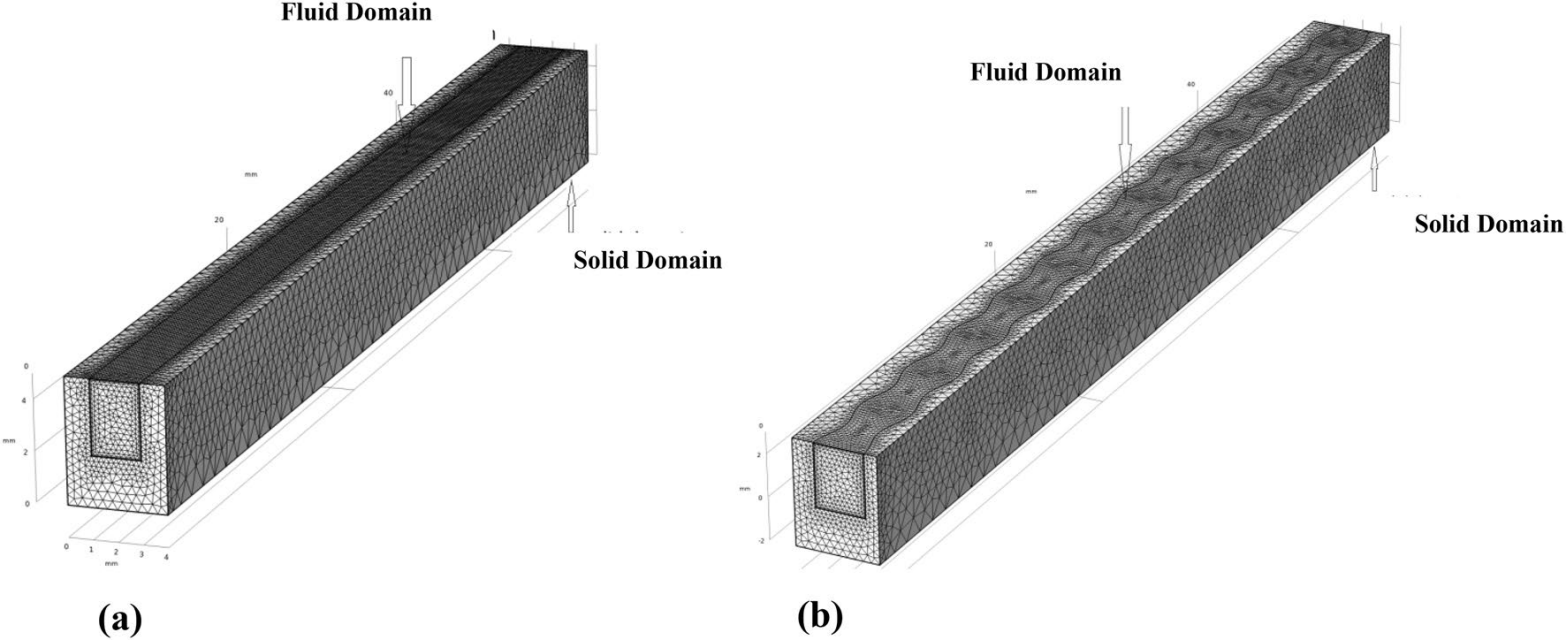
Mesh distribution of a computational domain (**a**) conventional channel (**b**) wavy channel.

To guarantee that the mesh grid is accurate, the examination was carried out by taking a number of different mesh types (normal, fine, finer and extremely fin) for rectangular mini channel (Fig. 2). Types of mesh tested and explained in Table 2, each type contains the number of elements domains, boundary elements and edge elements by computing the average Nusselt number in hot surface contact between solid and fluid domain as dependent variable since the Nusselt number is a global parameter, Fine mesh was selected for having minimum error. The Nusselt number and maximum temperature (Tmax) have been assessed for each mesh for conventional minichannel heat sink at Reynolds number Re 400 and heatflux 180 kW/m^2^. The relative error of the chosen parameterss was calculated using the equation below^35^

**Table 2:** Mesh sensitivity test for rectangular mini channel heat sink at Re = 400, q = 180 kW/m^2^.

Element size	Domain element	Boundary element	Edge element	Nu	Nu_error%	T_max_ (K)	T_max_-error%
Extra Fine	7,820,763	272,224	3843	10.060	18.83%	343	2.62%
Finer	1,876,205	92,486	2272	12.394	17.3%	334	1.79%
Fine	570,155	38,210	1475	14.994	1.42%	328	0.304%
Normal	222,511	20,322	1065	15.211	–	327	

18 $$ {\text{E}}\left( \%  \right) = \left| {\frac{{Z_{2}  - Z_{1} }}{{Z_{1} }}} \right| \times 100 $$

where Z signifies any parameter; such as Nusselt number, pressure drops, friction factor, and temperature, Z1 and Z2 mean the variable values obtained from the finest grids as well as other grids^29^. Table 2 illustrates this. The solution's mesh independence was ensured by the 'fine' mesh, which allowed for the optimal runtime. Two modeling models of previous cfd analysis were compared to ensure the precision of the data. The first model was associated to^14^ who executed a numerical analysis to investigate heat and flow in the rectangular heatsink (MCHS) fitted with coil inserts using (FVM) based software—ANSYS CFX I (www.ansys.com). Figures 3 and 4 display a comparison of the current COMSOL 5.6 (www.comsol.com) simulations with the available calculations, which a good agreement is seen between the two approaches. The another validation was with^7^ Who accompanied a numerical investigation to explain the influence of a corrugate channel structure and nano fluid on the application of a heat transfer characteristics of a micro channel heat sink. With this analysis, the validation was carried out and good consensus was seen in Figs. 5, 6, 7 and 8.

**Figure 3:**
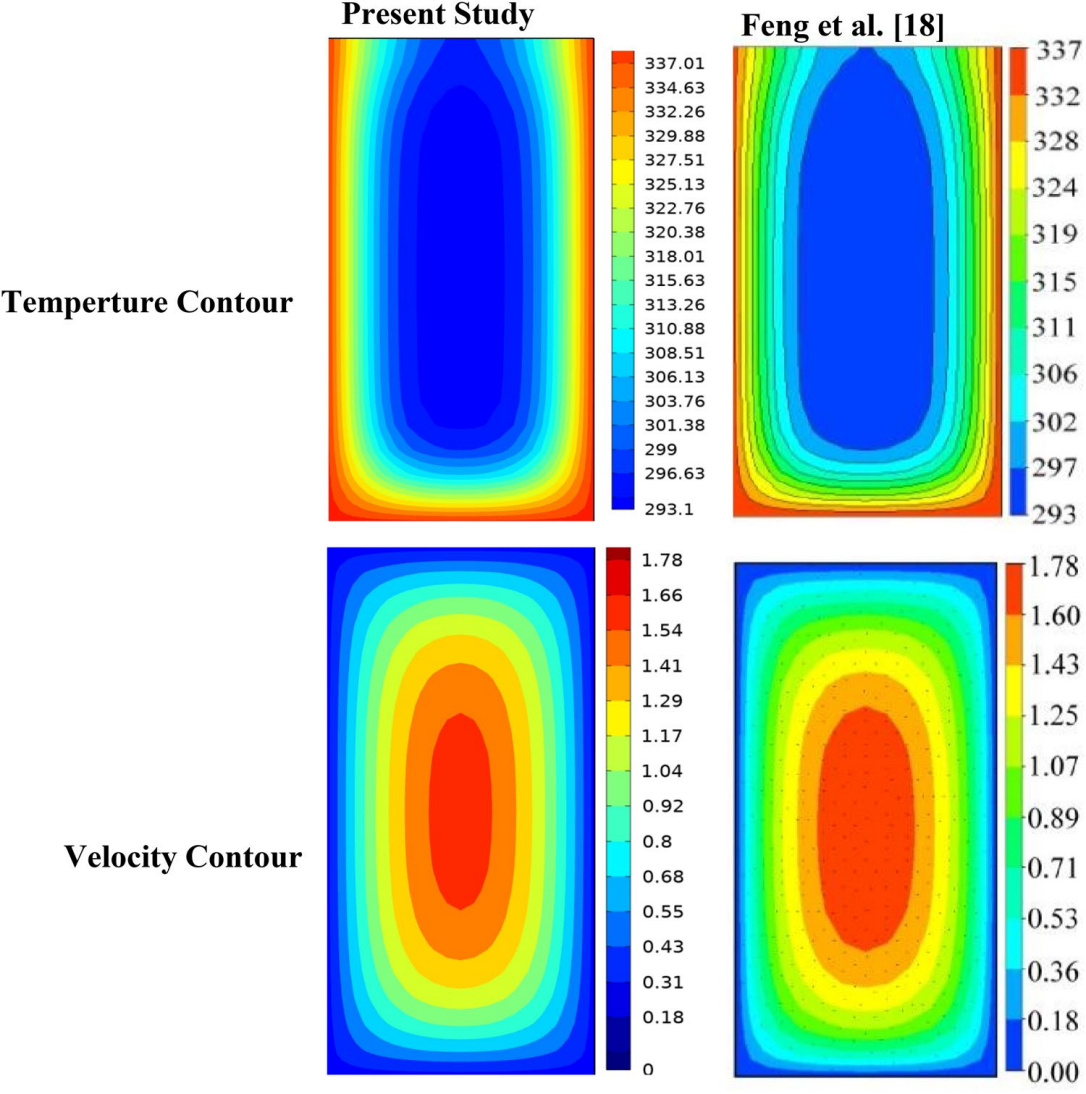
Temperature and velocity contours comparsion between present work and Feng et al.^14^ on a cross section (x/L = 0.625) in microchannel at Re = 663 and qw = 400 kW/m^2^.

**Figure 4:**
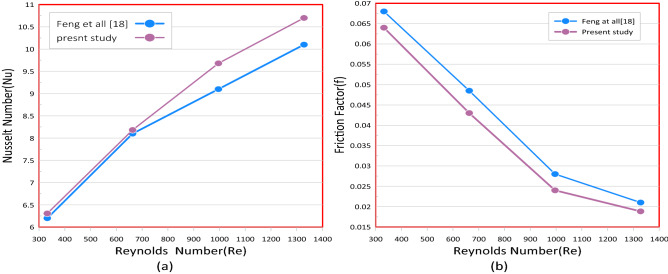
Code validation with Feng et al.^14^ by comparsion of variation of Nusselt number (**a**) and friction factor with Reynolds number (**b**).

**Figure 5:**
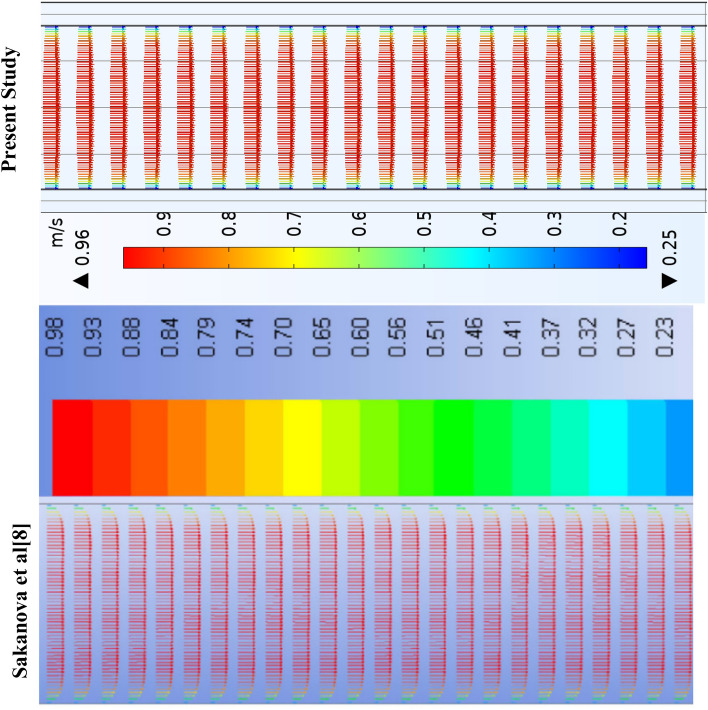
Comparsion of velocity vector of rectangle channel along z-axis with Sakanova^7^.

**Figure 6:**
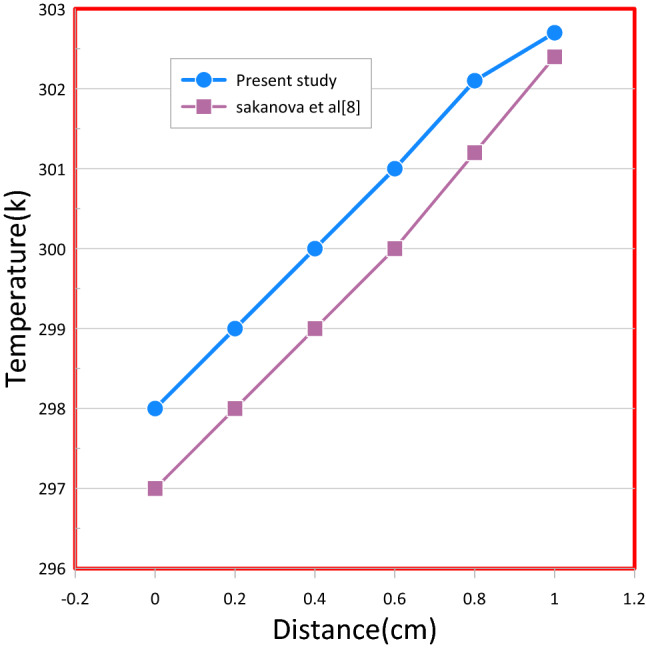
Comparison of the current investigation to Sakanova et al's temperature distribution path through the rectangular channel.

**Figure 7:**
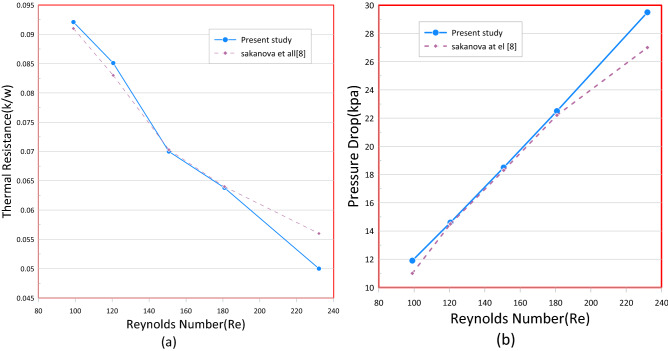
(**a**) Thermal resistance and (**b**) pressure drop dependence on Re for rectangular MCHS using pure water.

**Figure 8:**
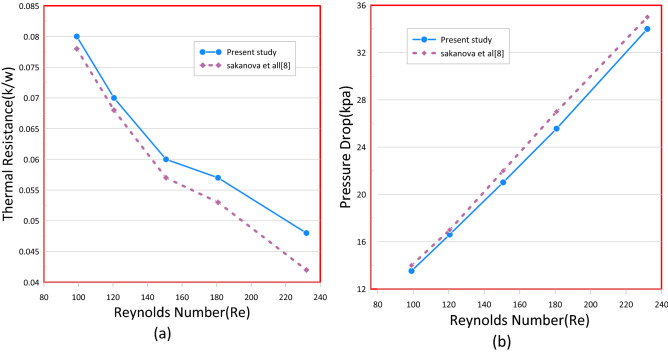
(**a**) Thermal resistance and (**b**) pressure drop dependence on Re for rectangular MCHS using diamond (5%)–water nanofluid as the coolant.

## Result and discussion

### Hydrothermal performances features of the mininchannel heat sink

The distribution of a heat and fluid flow in the mini-channels must be studied because it has a direct effect on the cooling efficiency.

#### Velocity contour characteristics

**Figure 9:**
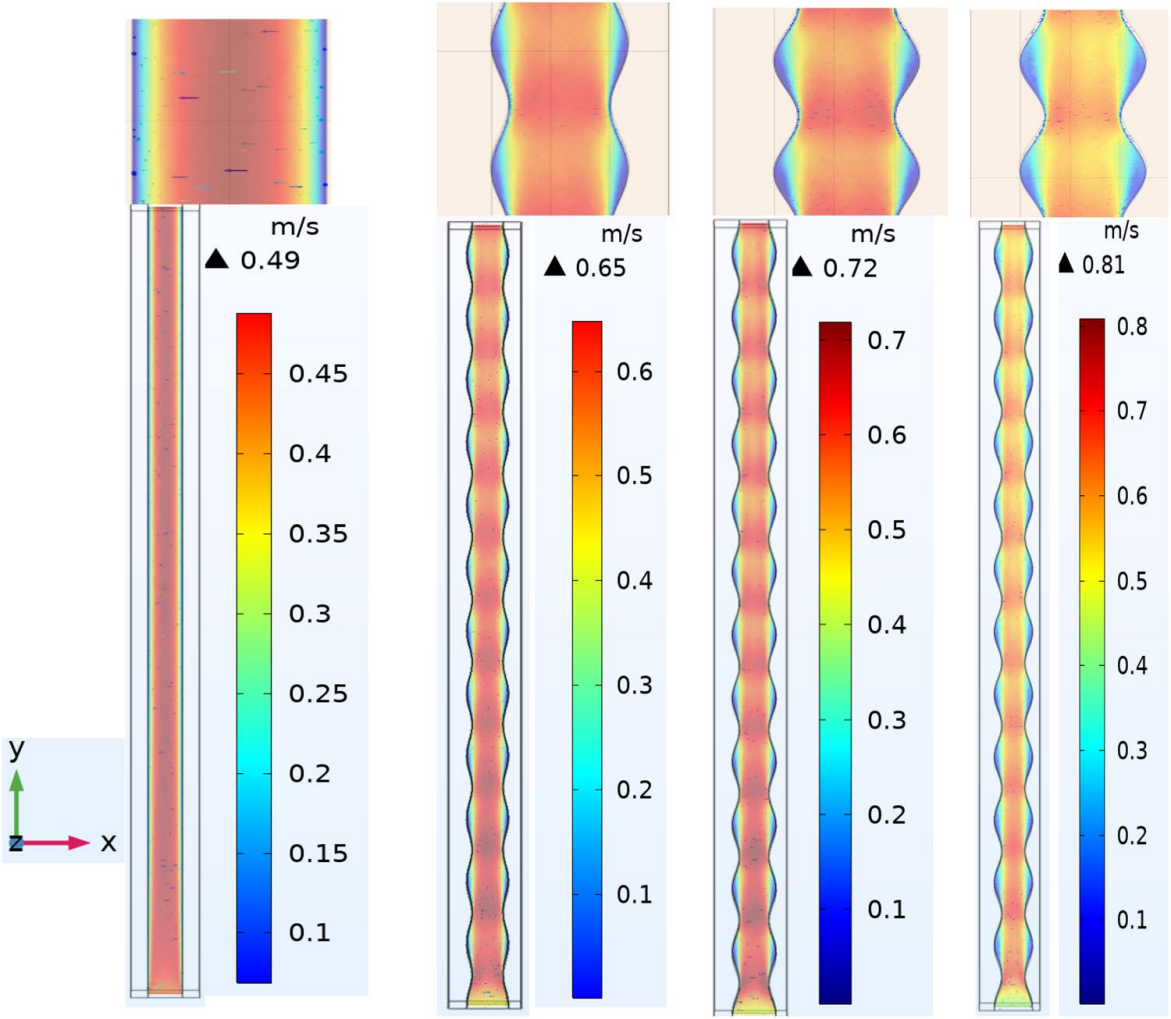
Effect of corrugtion walls on velocity magnitude at φ = 0.075, Re = 800, q_w = 180 kw/m^2^ for amplitude (A) = 0, 0.15, 0.2 and 0.25.

Figure 9 demonstrates that the velocity distribution and magnitude for a rectangular and a wavy channel with different amplitude along y-axis (A = 0, 0.15, 0.2 and 0.25) with Reynolds number Re = 800 and Ag (7.5%) as a coolant. Because of its wavy structure, the velocity of the fluid will increase with the increase in the amplitude of the wave, as it reached in the rectangular channel (0.49 m/s) and in the wavy shape it reached (0.65, 0.72, 0.81) m/s according to the wave amplitude (0.15, 0.2, 0.25) mm respectively, where the wavy shape gives an activity and an additional boost to the kinetic energy, as a result of reducing the adjacent layer in the waist area which causes an increase the fluid flowing mass thus increases the kinetic.energy of the fluid. The max velocity in the y direction is moved from the centerline to the curvypeaks and the flow next to wavy peaks is accelerated. Therefore, the hydrodynamic and thermal edge layers are becoming thinner. There is thus an improvement in the heat transfer coefficient.

#### Temperature contour characteristics

**Figure 10:**
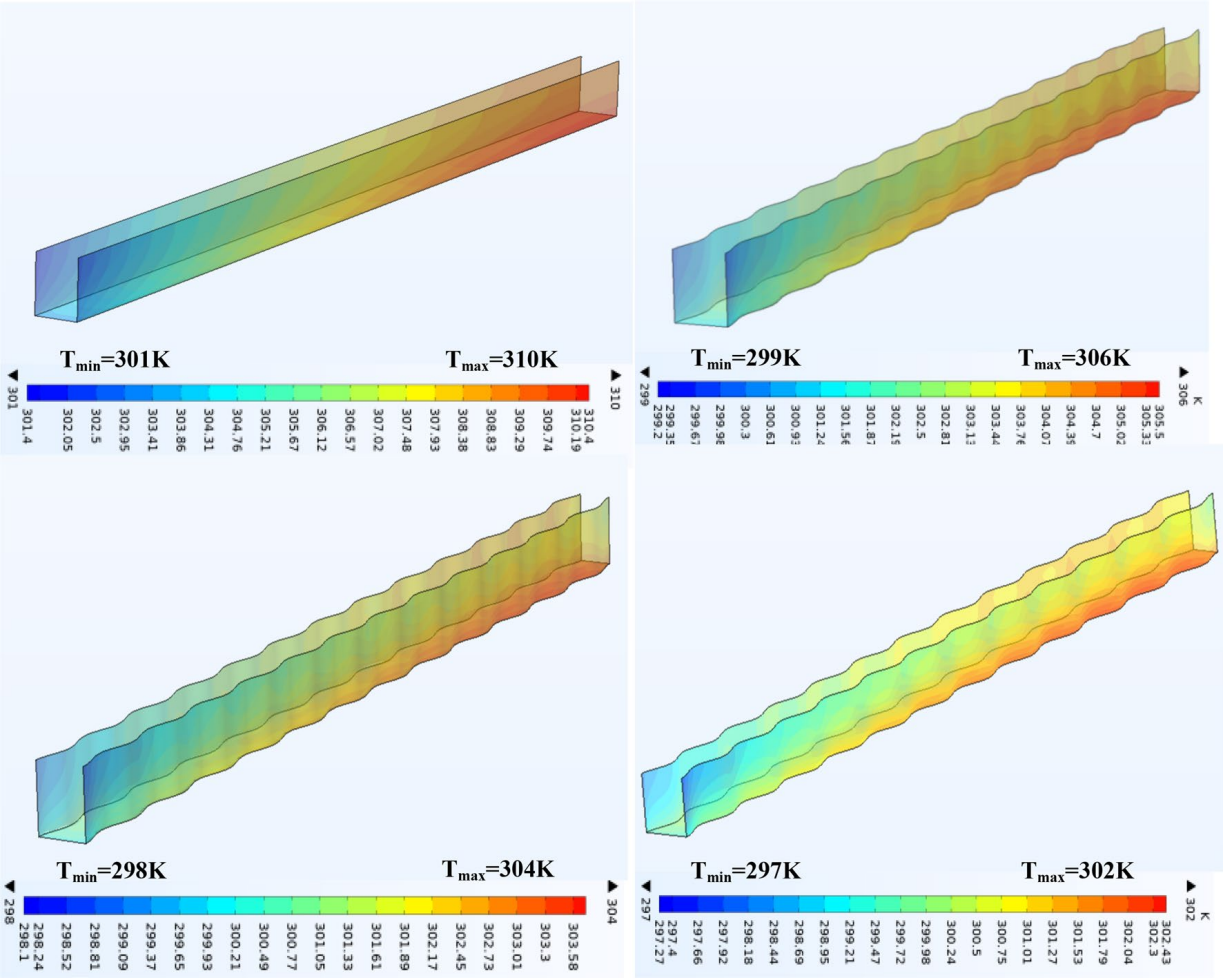
Effect of corrugtion walls on temperature distribution at φ = 0.075, Re = 800, q_w = 1.85E5 w/m^2^ for amplitude (A) = 0, 0.15, 0.2 and 0.25.

To explain a distribution of temperature along the interaction surfaces between the fluid and solid in various shape of minichannel heatsink (Ag) nano fluid was selected, at Reynolds number Re = 800 and volume concentration of nanofluid (0.075) under the same inlet conditions (Tin = 293 K) as shown in Fig. 10. The temperature rises from the inlet to the exit along the y-direction for various channels a maximum heat dispersal from a mini channel heat sink take place at inlet because of a maximum temperature variance between a channel and inlet fluid temperature. As the process of heat transfer occurs by convection between the channel's walls and the fluid flowing through it, as well as arise in heat transfer based, on the amount of the variance in temperature between a surface and a fluid, where the highest heat transfer occurs at start of the current because the fluid's temperature is small and therefore a variance in temperature is high and thus increases The transfer of heat from a surface to cooling fluid, thus increasing its temperature during its flow in the stream, and consequently the difference in temperature decreases when the stream is depleted and the heat transfer decreases. Therefore, we notice the increase in temperature along the Y axis. The highest mini-channel heat sink temperature with the wavy-channel is lower than that of a rectangular channel and it decrease with the increase of wavy amplitude (A) as shown in Fig. 10 for reasons we will discuss them in the section “Effect of a wavy wall of MCHS”.

### Effect of a wavy wall of MCHS

**Figure 11:**
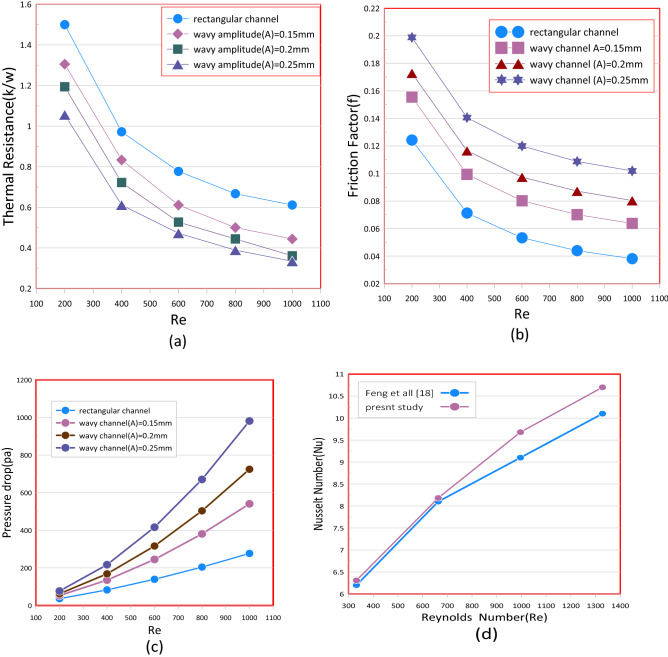
Relation between (**a**) thermal resistance (R_th_) (**b**) friction factor (f) (**c**) pressure drop and (**d**) Nusselt versus Reynolds number (Re) in rectangular channel and wavy channel.

To evaluate the effects of the wavy wall of mini channel, distilled water has been used at first as cooling fluid. Figure 11 demonstrations the thermal-resistance, friction-factor, and pressure drop against Reynolds number for both a conventional and all wavy mini channel heat sink. From Fig. 11a, it displays that the relationship of thermallresistance )is the resistance shown by the component to the transfer of heat by conduction through its thickness and increasing it means increasing the element's ability to heat insulation( and Reynolds number (Re) are reverse proportionality. The thermal resistance reduces as the Reynolds number rises because of an increased flow rate and thermal dispersion effects. This is apparent that in all these the cases (A) = 0.25 mm have a minimum thermal resistance. It is also clear that rectangular channel thermal resistance is greater than all wavy channels. The comparison between a wavy and rectangle channel shows a major difference in the efficiency of the heat transfer. From Fig. 11b, It indicates the reversely proportional ratio of friction factor (f) and Reynolds number (Re) As the Reynolds number increase, a friction factor decrease. The lowest friction factor value in a rectangular channel can be seen Also, it is obvious that the friction factor (f) of a wavy channel at wavy amplitude (A) = 0.25 mm is greater than all wavy cases. These effects are as a result of a rise in drag force that a result of reprocessing a flow and changing its path inside the wavy-channel. The friction factor (f) increase with the increases in wave amplitude as a result of the high pressure reduction persuaded by increase the flow objection with a wavy path.

From Fig. 11c, the relationship between a pressure drop and Reynolds number (Re) can also be found to be proportional. With an increase in the Reynolds number (Re), a pressure drop also increases. It is clear that the corrugate channel has a greater pressure drop than a conventional channel. with high wave amplitude, the more the pressure drops accure because of flux disruptions due to variations in the orientation of the channel and secondary flow in a wavy channel created and the contacts between a vortices and the walls of the channel often increase heat sink pressure losses.

Figure 11d shows the Nusselt number ratio of rectangular mini channel and wavy channel for various cases. The heat transfer in any wavy channel relative to a rectangular channel can be expected to be increased. Regardless of a channel configuration, the (Nusselt number) rises with increasing the Reynold number at a cost of higher pumping power Pp. With the Reynolds rise, a thermal boundary layers in the channels decreases with the temperature gradient increasing near to channel walls, thereby contributing to higher heat transfer. Fluid flow through waves is subject to a centrifugallforce which disrupts a flow field and which can result in a recirculation of fluids^27^.

The secondary flow from the channel moving the liquid from the center part of this channel to its hot wall and from areas adjacent to a channel walls to the center part of the channel is inducted by the fluid recirculation, thus the force of a secondary flow, rises when the Reynolds number is increased. Furthermore, variations in the curvature in a wall of a channel cause changes in the orientation of a vortices in a channel. As a result of a fluid being intercepted by a wavy path during its flow, it makes it more effective in transmitting movement, as the areas of narrowing of the wave act as an accelerator, followed by areas where the area of flow increases. This sequence creates a kind of stratification between the layers of the flowing fluid that makes it more effective. The improvement of the thermal efficiency in a heat sink with wavy channels is due to an increase in a region of heat transfer and formation of a secondary-flow vortices that intensify convectio. As an improvement of heat transfer is induced in two parts, namely, increasing the contact area between the surface of a heat transfer and the cooling fluid, and the other part is changing the direction of the flow continuously between directing the flow inward in the narrowing areas, followed by directing the flow outward towards the side walls in the expansion areas, and this direction occurs periodically down to the end of the stream.

### Nanofluid effect in a wavy mini-channel heat sink

**Figure 12:**
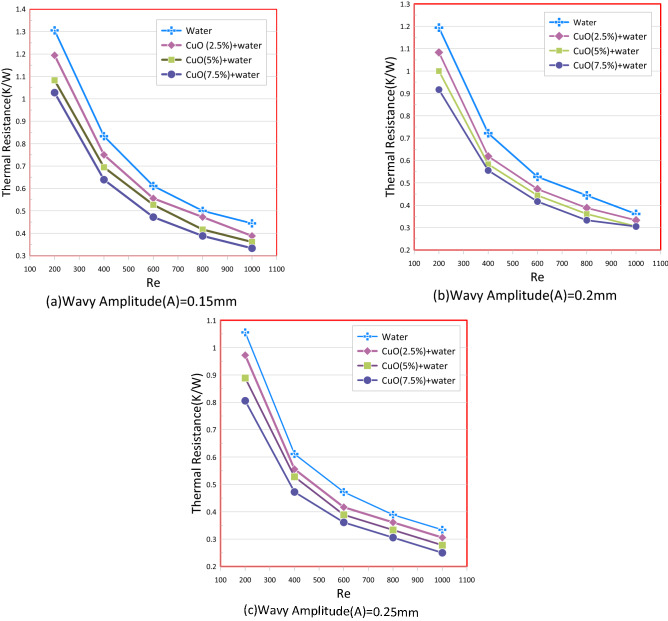
Thermal resistance versus Reynolds Number for wavy channel: (**a**) Wavy Amplitude (A = 0.15 mm). (**b**) Wavy Amplitude (A = 0.2 mm). (**c**) Wavy Amplitude (A = 0.25 mm).

In Fig. 12 the thermal resistance of the wavy channel with (CuO) nanofluids and water as a coolant is seen to decrease when the Reynolds number rises. Also, with the fraction of nanoparticles increased, thermal resistance was further reduced. This decrease in thermal resistance could be interpreted by the greater the nanofluid thermal conductivity than pure water. This is because of the increase of the thermophysical properties owing to the addition of thermo-conductive nanoparticles into water as a basis fluid.

**Figure 13:**
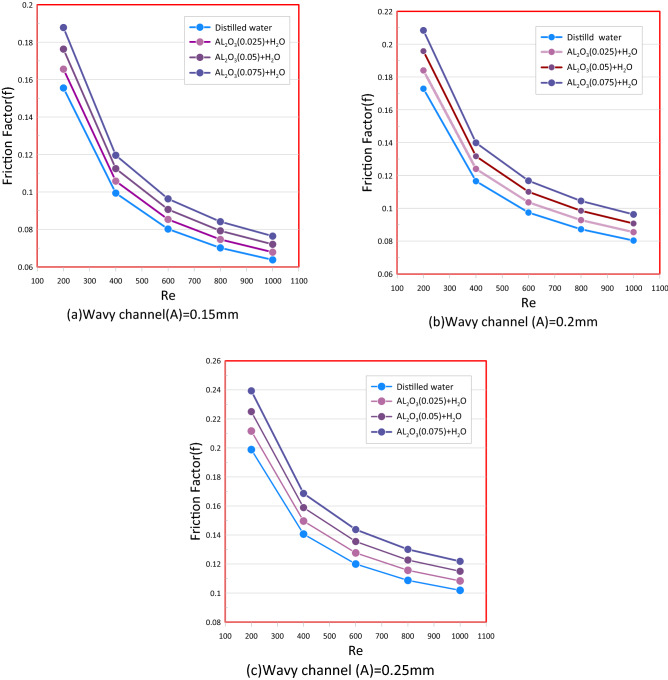
Friction Factor versus Reynolds Number for wavy channel: (**a**) Wavy Amplitude (A = 0.15 mm). (**b**) Wavy Amplitude (A = 0.2 mm). (**c**) Wavy Amplitude (A = 0.25 mm).

In the Fig. 13 the AL_2_O_3_ nanofluid with various concentrations volume (φ) was selected to illustrate the effects of the nanofluid on the friction factor and it was found that the friction factor is greater for nanofluides and rises in tandem with the rise of the wave amplitude and concentrations volume (φ) and decrease with increase the Reynolds number. It's also worth noting that, instead of pure fluid, you might useing nanofluid as a coolant causes an additional rise in pressure drop in comparison to distilled water due to the presence of nano solid particles in nanofluid that cause an increase in nanofluid viscosity and density which causes an increase friction factor. the friction factor decreases by increasing the Reynolds number because a friction factor and speed have reverse relationship.

**Figure 14:**
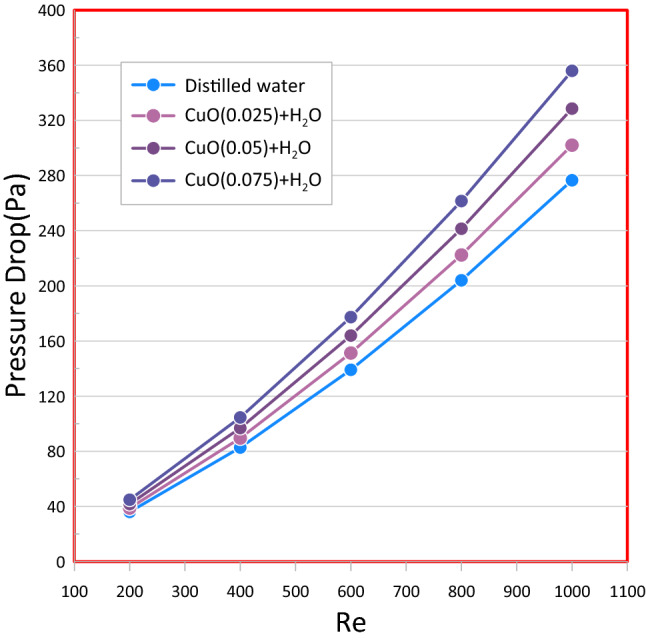
Show the effect of used nanofluid as acoolant on pressure drop.

Figure 14 represents the pressure drop versus Reynolds number which show that higher pressure losses are correlated with the rise in heat transfer efficiency of a heat sink achieved using nanofluids as compared to the standard fluid, This is because of a greater viscosity of nanofluid. With increasing nanoparticle volume fraction, interactions between nanoparticles increase that leads to a rise in the actual nanofluids' viscosity. Therefore, the pumping power necessary for driving a coolant in a heatsink rises with the rising nanoparticles volume fraction.

### The effect of nanoparticles type in wavy-channel

**Figure 15:**
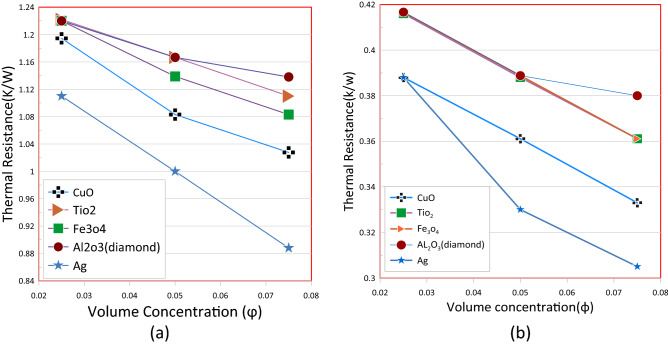
Overall thermal resistance versus volume concentration of various types of nanofluid (**a**) Re = 200, (**b**) Re = 1000.

Wavy channels are numerically examined for three dimention heat transfer using different nano-fluids, such as (Al_2_O_3_, CuO, TiO_2_, Fe_3_O_4_, and Ag) water nano-fluids as a coolant. The volume concentration range is between 0.025 and 0.075 percent and Reynolds number between 200 and 1000. For the influence of the nanoparticle concentration volume on the total thermallresistance, the case of high and low Reynolds are chosen. Based on the outcome from Fig. 15. In general, all types of nanoparticles move in the same direction, which is the decrease in thermallresistance with a rise in the ratio of the concentration of nanomaterials. In Fig. 15a and b, it has also been found that a thermal resistance decreases by increasing the numbers of Reynold. The Ag nano fluid showed the lowest thermal resistance value in all the concentrations and for both Reynolds number The gross thermal resistance decrease as a volume fraction of nanopartical increases The nanofluid (Ag-water) has the lowest thermal resistance compared with other forms of nanofluid because (Ag–water) nanofluid has a better thermal conductivity. As follows, the influence of nanofluids can be explained. Both thermallconductivity and dynamicc viscosity are improved by the presence of nanoparticles in base fluids, followed by a reduction in heat capacity. It is also possible to increase the convective heat transfer coefficient by increasing thermal conductivity. At the same time, dynamic viscosity increases and heat capacity decrease, causing the mean velocity of nanofluids to decrease.

**Figure 16:**
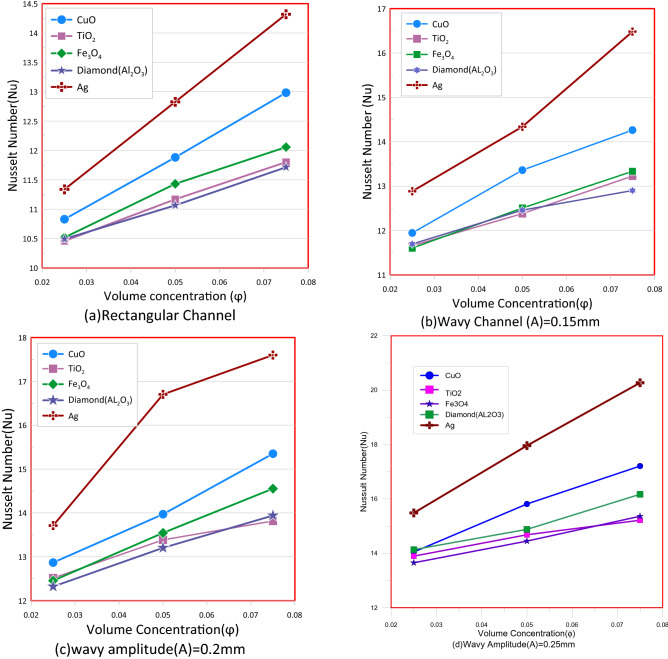
Show Nusselt number (Nu) versus Volume concentration for different channels.

**Figure 17:**
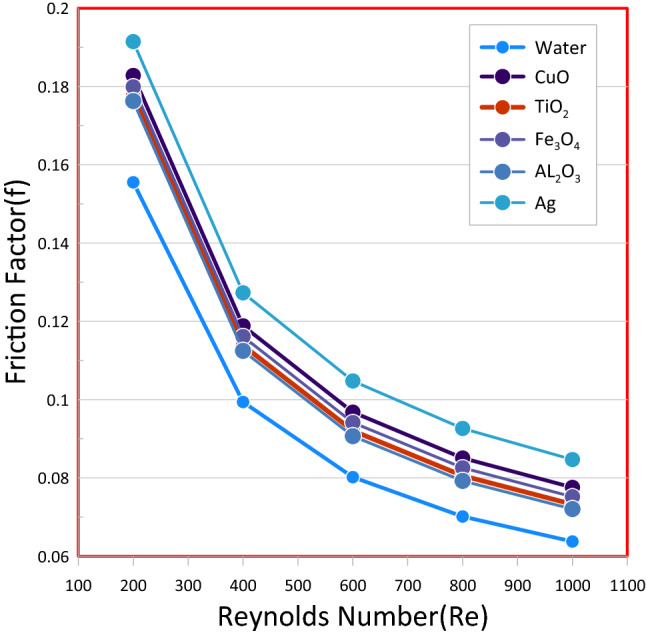
Friction factor of the wavy channel (A = 0.15 mm) and volume concentration (ϕ = 5%) for various nanofluids with Reynolds Number.

Figure 16 show the influence of concentration volume on the Nusselt number for different shape of the channel at Reynolds Number (Re = 200). In situations where nanofluids are used as coolants, the Nusselt number (Nu) is greater than the one using water when cooling the heat sink. This is primarily attributed to higher thermal efficiency of nanofluids in contrast to a base fluid, This leads to an increase in the heat conduction contribution to the total energy transmission and it increases with increasing proportion of the volume volume of a nanoparticles. It's that attributable to increased overall area of heat transfer between nano particle and a base fluid, and the increase in the rate of collision of nanoparticle that increases the particle's Brownian motion, causing in an increase in the mixture's effective thermal conductivity^27^. Figure 17 shows the effect of nanoparticles type on friction factor which Ag has the maximum and Al_2_O_3_ has the minimum values for f.

### Temperature variance with the flow of fluids in the mini-channel

**Figure 18:**
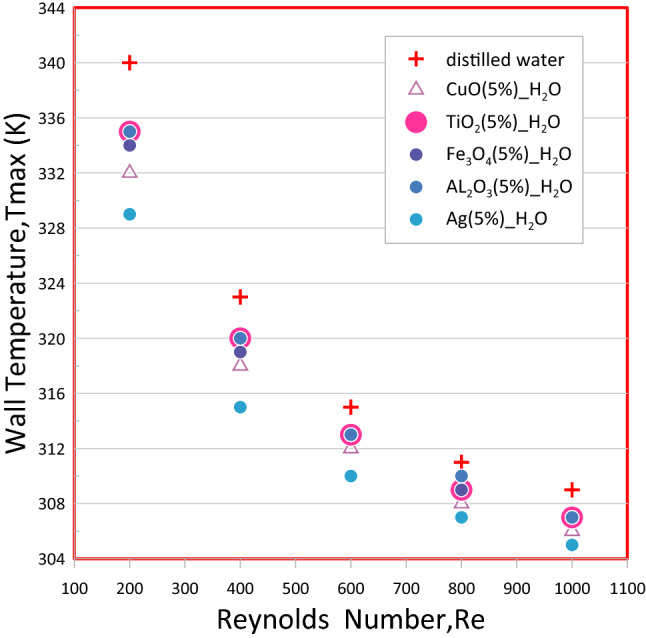
shows the maximum temperature of mini channel for different nanofluid at wavy channel (A = 0.15 mm) for the different Reynolds number (Re).

Figure 18 shows the max temperature for different working fluids; distilled water, Al_2_O_3_–H_2_O, TiO_2_–H_2_O, CuO–H_2_O, Fe_3_O_4_–H_2_O, and Ag–H_2_O nano fluids are considered as the coolants in the Wavy channel (A) = 0.15 mm for the different Reynolds number (Re). With increased velocity, the temperature of a mini-channel wall drops. Because of Newton's law of cooling, the heat transfer coefficient has an inverse relationship with temperature variation.

19 $$Q=h.A.\nabla T $$

where, h: heat transfer coefficient (w/m^2^ k), A:surface area (m^2^), $\nabla T$: temperature variation (k).

**Figure 19:**
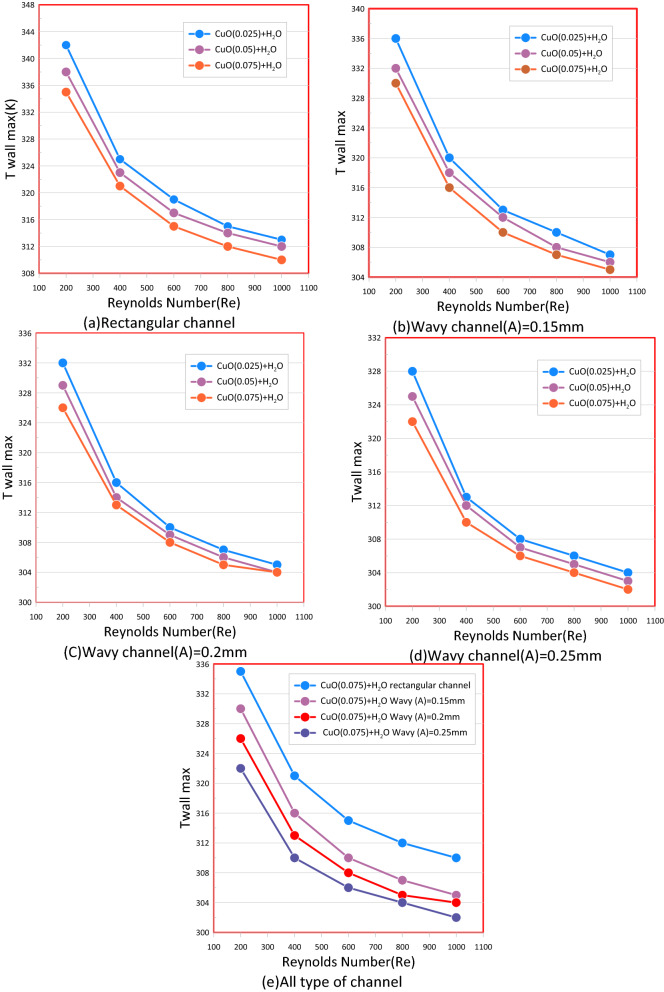
Show the relation between the max wall temperature and a Reynolds number of different types of mini channel heat sink.

The Ag–H_2_O nanofluid, which has the largest heat transfer improvement compared to otherr nanofluids, showed the lowest temperature value on a mini-channel walls, as shown in Fig. 18. To illustrate the effect of volumetric fractures on the temperature of the walls for the various studied channels the (CuO–water) with different volume concentrations was selected As shown in Fig. 19 for rectangular (a) and wavy mini channel at different wavy amplitude (A = 0.15, 0.2, 0.25) mm at (b), (c)and (d) respectively. It showed that if the nanoparticle volume fractions (φ) increase the wall temperature decrease. This is because of a rise in overall heat transfer area between the particles and a base fluid, and an improvement of nano particle impact rate that increases the Brownian motion of particles causing in the improvement of the thermal conductivity of the mixture.

### Performance evaluation criterion (PEC)

The Combined influence of Nusselts number and the friction factor was employed to measure the overall hydrothermallbehavior of wavy channel using PEC as shown in Eq. (20)^27^

20 $$PEC=\frac{(Nui/Nuo)}{{(fi/fo)}^{1/3}}$$

where subscript (o and i) represents a conventional heat sink and wavy mini channel.

**Figure 20:**
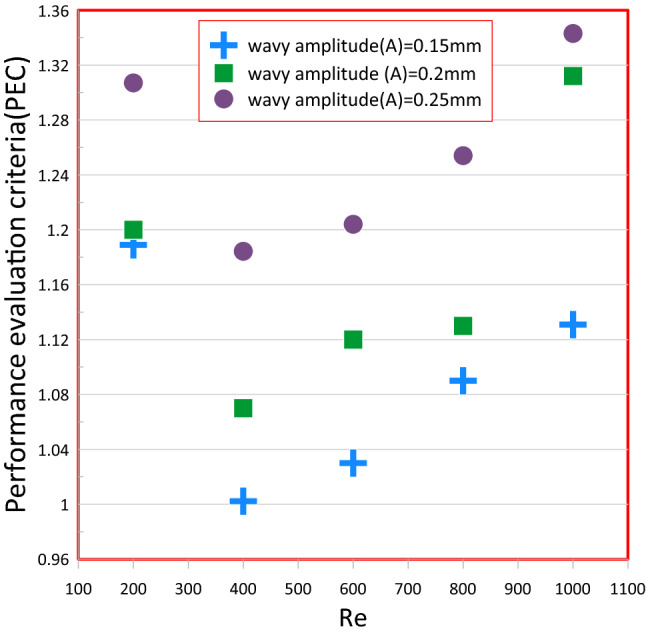
Variation of the thermallperformance factor versus Reynold number for wavy channel with various wavy amplitude (A) at distilled water as a coolant.

Figure 20 depicts the relation between a thermal performance factor and a Reynold number for a wavy channel with different wavy amplitude (A) and distilled water asa working fluid. In this analysis, It is clear to see that a thermal efficiency factor greater than unity over the entire Reynold number range and almost increase with an increase in Reynold number. Additionally, when a wave amplitude increases, the thermal performance factor rises, This implies that the increase in pressure loss can be balanced by an improvement in the transfer of heat when using of Wavy mini channels comparison to rectangular mini channels.

## Conclusions

In a present study, heat transfer was enhanced through two methods, one of which is low-cost, which is by corrugating the walls, and the other cost-effective method is the use of different nano fluid, and that the two methods have proven their effectiveness in increasing the rate of heat transfer through proven results. Where a numerical analysis between rectangular and wave mini-channel heat sink and fluid flow characteristics was conducted using five different nanofluids, namely Al_2_O_3_–H_2_O, Copper oxide (Cuo–H_2_O),Titanium Oxide (TiO_2_–H_2_O), Iron Oxide (Fe_3_O_4_–H_2_O) and silver (Ag–H_2_O). It investigates the impacts of a wave amplitude, Reynold number and volume fraction of various nanofluids. If water is used as a coolant, the conventional rectangular mini-channel cooling efficiency is overcome by the wavy minichannel heat sink. Furthermore, the greater the amplitude of a wavy-channel, the less thermal resistance delivers additional pressure drop. In this research, the performance of five kinds of nanofluids was examined. All of them demonstrate the better application of cooling compared to distilled water and the existence of nanofluids, however, increase the drop in pressure and the friction factor. The Ag–H_2_O nanofluid outperforms all other nanofluids by having a highest heat transfer coefficient and the lowest thermal resistance. Replacing the conventional channel with a wavy channel in a heatsink results in an increasee of 28–52% in Nusselts number.

## Data Availability

The datasets used and/or analysed during the current study available from the corresponding author on reasonable request.

## List of symbols

A: Amplitude of wavy
Cp: Specific heat at constant pressure (KJ/kg K)
D_h_: Hydraulic diameter (m)
*f*: Fanning friction factor
h: Heat-transfer coefficient(W/km^2^)
H, H_c_: Height of the channel and channel
k: Thermal conductivity (W/m K)
L: Length of the heatsink (m)
N: Undulation number
Nu: Nusselt number
Pr: Prandtle number
u_in_: Inlet velocity (m/s)
∇p: Pressure drop (Pa)
p: Pressure (Pa)
q_in_: Heat flux (W/cm^2^)
Q: Total heat flux
*R*_*th*_: Total thermal resistance(K/W)
*Re*: Reynolds number
*t*: Thickness of the heatsink
*T*: Temperature (K)
*u**, *v**, *w**: Velocity of flow (m/s)
W, W_c_: Width of the heatsink and channel
X, S: Horizontal and vertical distance (m)
x, y, z: The Cartesian-coordinates (m)

## Greek symbols

μ: Dynamic-viscosity (kg s/m)
$$\rho $$: Density (kg/m^3^)
$\varphi$: Nanoparticle volume fraction

## Subscripts

Ch: Channel
FVM: Finite volume method
*f*: Fluid
in: Inlet
nf: Nano fluid
s: Solid domain
p: Solid particles
max: Maximum

## Abbreviations

Cond.: Conduction
Conv.: Convection
